# Exploring the fabrication and transfer mechanism of metallic nanostructures on carbon nanomembranes via focused electron beam induced processing

**DOI:** 10.3762/bjnano.12.26

**Published:** 2021-04-07

**Authors:** Christian Preischl, Linh Hoang Le, Elif Bilgilisoy, Armin Gölzhäuser, Hubertus Marbach

**Affiliations:** 1 Physikalische Chemie II, Friedrich-Alexander Universität Erlangen-Nürnberg, 91058 Erlangen, Germany; 2 Fakultät für Physik, Universität Bielefeld, 33615 Bielefeld, Germany

**Keywords:** 2D materials, carbon nanomembranes (CNMs), focused electron beam-induced processing, metallic nanostructures, self-assembled monolayers

## Abstract

Focused electron beam-induced processing is a versatile method for the fabrication of metallic nanostructures with arbitrary shape, in particular, on top of two-dimensional (2D) organic materials, such as self-assembled monolayers (SAMs). Two methods, namely electron beam-induced deposition (EBID) and electron beam-induced surface activation (EBISA) are studied with the precursors Fe(CO)_5_ and Co(CO)_3_NO on SAMs of 1,1′,4′,1′′-terphenyl-4-thiol (TPT). For Co(CO)_3_NO only EBID leads to deposits consisting of cobalt oxide. In the case of Fe(CO)_5_ EBID and EBISA yield deposits consisting of iron nanocrystals with high purity. Remarkably, the EBISA process exhibits a strong time dependence, which is analyzed in detail for different electron doses. This time dependence is a new phenomenon, which, to the best of our knowledge, was not reported before. The electron-induced cross-linking of the SAM caused by the cleavage of C–H bonds and the subsequent formation of new C–C bonds between neighboring molecules also seems to play a crucial role in the EBISA process. Previous studies showed that iron nanostructures fabricated on top of a cross-linked SAM on Au/mica can be transferred to solid substrates and grids without any changes, aside from oxidation. Here we demonstrate that iron as well as cobalt oxide structures on top of a cross-linked SAM on Ag/mica do change more significantly. The Fe(NO_3_)_3_ solution used for etching of the Ag layer also dissolves the cobalt oxide structures and causes dissolution and reduction of the iron structures. These results demonstrate that the fabrication of hybrids of metallic nanostructures onto organic 2D materials is an intrinsically complex procedure. The interactions among the metallic deposits, the substrate for the growth of the SAM, and the associated etching/dissolving agent need to be considered and further studied.

## Introduction

Focused electron beam-induced processing (FEBIP) is a powerful maskless “direct-write” approach for the fabrication of arbitrarily shaped nanostructures [1–5]. The most prominent method within the FEBIP family is electron beam-induced deposition (EBID). In EBID, a focused electron beam is used to locally dissociate adsorbed precursor molecules. Thus, a localized deposit of the non-volatile decomposition products forms, while the volatile fragments are pumped off [1–3]. By applying the EBID process it is possible to directly fabricate metallic nanostructures with arbitrary shape and size. Thus, EBID enables the fabrication of well-defined nanostructures [6–8]. Furthermore, the large amount of available precursors allows for the deposition of many different materials [3]. One major challenge in EBID is the undesired co-deposition of carbon and other impurities [4]. The resulting carbonaceous deposits need to undergo further purification steps in order to obtain satisfying results [4,9]. However, for certain precursors, EBID yields clean deposits when carried out under ultrahigh vacuum (UHV) conditions. It was shown that in UHV, for some precursors, an autocatalytic growth (AG) process occurs already at room temperature, which leads, upon further precursor dosage, to the dissociation of the precursor molecules on top of the initial EBID deposit. In the case of Fe(CO)_5_ this AG process results in the formation of deposits consisting of pure iron [10]. A second method from the FEBIP family, namely electron beam-induced surface activation (EBISA), also largely exploits the AG process [11]. In EBISA, the surface is, in a first step, irradiated and chemically modified without precursor dosage. In a second “development” step, a well-defined deposit is formed at the chemically activated sites, as the precursor is dosed and dissociates locally. Of course, for EBISA a suitable combination of substrate and precursor is a prerequisite, that is, the substrate must be chemically altered by the electron beam and the precursor molecule must be susceptible to the altered site. Substrates that are known to fulfill the prerequisite are silicon oxide [10,12], rutile TiO_2_(110) [13], thin layers of porphyrin molecules [14–15], and surface-anchored metal-organic frameworks (SURMOFs) [16–17]. For oxide surfaces it is known that the activation mechanism is based on reactive oxygen vacancies, which are locally created by electron-stimulated oxygen desorption [18–19]. Whereas for organic and metal-organic substrates the activation mechanism is still not fully understood [14–17]. So far, EBISA was effective with the precursors Fe(CO)_5_ and Co(CO)_3_NO in UHV [12,14–18,20] and Co_2_(CO)_8_ in high vacuum (HV) [13]. The purity of substantial deposits in EBISA is mainly determined by the AG process of the used precursor. For Fe(CO)_5_ the formation of high-purity crystalline iron deposits is feasible [10–12]. An advantage compared to EBID is that in EBISA the growth of the deposit relies on the AG process only. Therefore, undesired electron proximity effects caused by secondary or backscattered electrons have minor influence on size and shape of the deposit. Recently, it could be demonstrated that FEBIP can be used to fabricate functional hybrid structures consisting of metallic nanostructures on top of organic 2D materials. The prototype of organic 2D materials used in this approach are ultrathin carbon nanomembranes (CNMs) [21]. CNMs are versatile 2D organic materials with high thermal [22] and mechanical [23] stability that can be produced by electron-induced cross-linking of aromatic self-assembled monolayers (SAMs) [24] and transferred onto arbitrary substrates or grids to obtain free-standing 2D membranes [25]. The specific choice of the self-assembling molecules determines the thickness, porosity, stiffness, and the mechanical/electrical properties of the resulting CNM [26–27]. The SAMs that are used for the fabrication of CNMs consist of aromatic molecules, which are chemically bound to a Au or Ag surface via either thiol [26] or carboxylic [28–29] anchor groups. Via FEBIP, the SAM can be functionalized with metallic nanostructures and subsequently be transformed into a CNM by electron-induced cross-linking. Afterwards, the resulting hybrid structure can be lifted off from the substrate and transferred onto bulk substrates, such as SiO_2_, or onto TEM grids in order to obtain a free-standing CNM with a metallic nanostructure on top. It was shown that the membrane is mechanically stable enough during the whole process and that the metallic nanostructures fabricated via EBID and Fe(CO)_5_ remain unchanged, besides oxidation. This result was achieved by using a SAM consisting of 1,1′,4′,1′′-terphenyl-4-thiol (TPT) on a Au substrate [21].

In this work, we want to gain more insight into the underlying processes yielding such hybrid nanostructures by investigating the fabrication and transfer on the example of a SAM of TPT on a silver substrate. Consequently, a different chemical etching process is needed for the lift-off process during the transfer. In the case of gold, an etching solution of KI/I_2_/H_2_O is used. Whereas in this approach, the Ag substrate is dissolved by a solution of Fe(NO_3_)_3_. It is important to study the effect of the underlying substrate and the associated effect on the resulting transferred hybrid structures as, for example, SAMs with carboxylic anchor groups can be fabricated on Ag [28–29]. In order to fabricate well-defined nanostructures it is also essential to study the different FEBIP techniques on SAMs. Therefore, we investigate EBID and EBISA with the precursors Fe(CO)_5_ and Co(CO)_3_NO on TPT SAMs bound to a Ag surface.

## Results and Discussion

**Figure 1:**
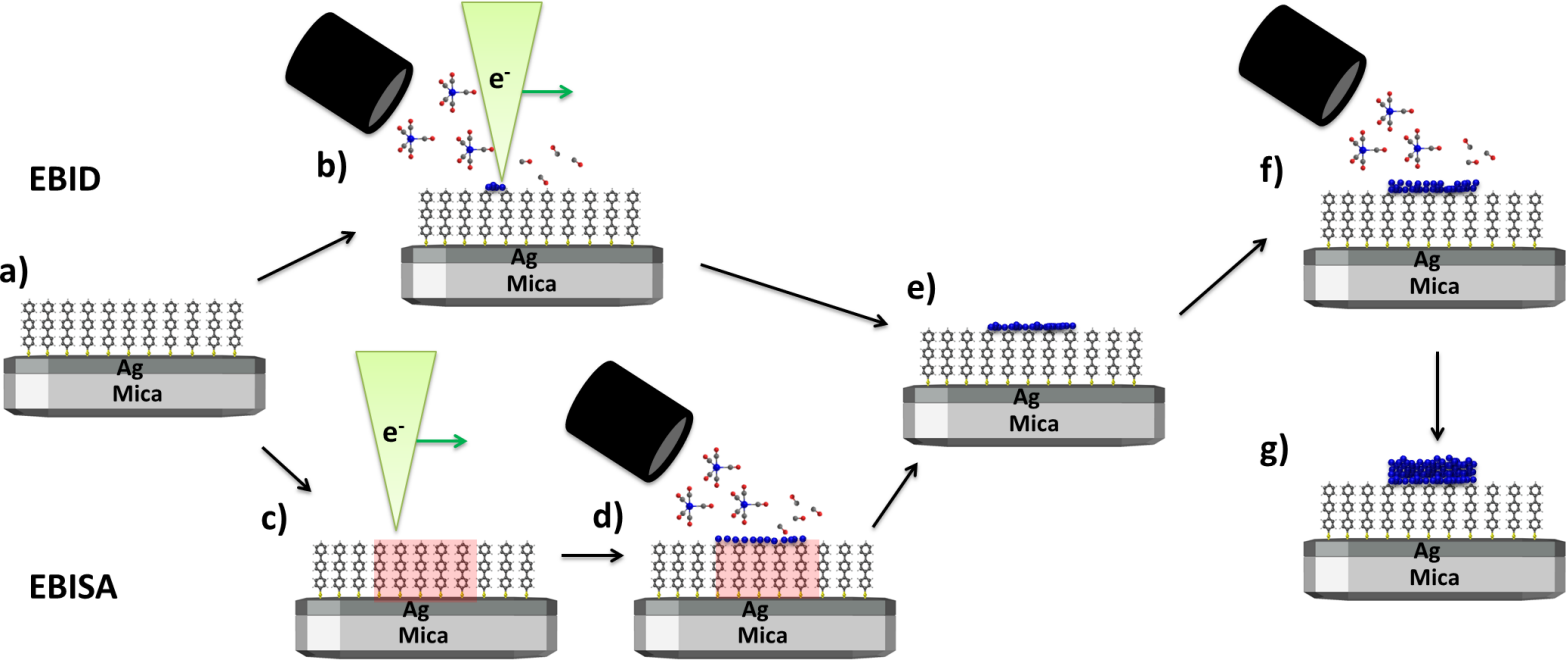
(a) SAM consisting of TPT molecules on a Ag(111)/mica substrate. (b) EBID process on the SAM Ag substrate. (c) Local activation of the TPT SAM with the focused electron beam within the EBISA process. (d) Precursor dosage after electron irradiation. If EBISA is possible the precursor dissociates locally on the activated sites. (e) Initial deposit that is formed by EBID respectively EBISA. (f) AG process that occurs on top of the initial deposit upon further precursor dosage. (g) Final well-defined nanostructure on top of a TPT on Ag(111)/mica sample.

First, the results of EBID and EBISA experiments and subsequent AG with both precursors on a TPT SAM on Ag(111)/mica sample will be discussed. The schematics of these experiments are depicted in Figure 1. For both methods a SAM consisting of TPT molecules bound via the thiol group onto a 300 nm thick Ag layer on mica was used (Figure 1a). In EBID, the precursor molecules are dissociated by the impact of the focused electron beam (Figure 1b). Whereas in EBISA, in a first step, the SAM is locally activated by the focused electron beam, indicated by the red area in Figure 1c. The formation of a deposit will only occur when these active sites cause the dissociation of the precursor molecules (Figure 1d). Assuming that the two described methods are effective, an initial well-defined local deposit is formed in both cases (Figure 1e). Upon further precursor dosage, the AG process occurs and leads to further agglomeration of material on top of the initial deposit (Figure 1f). If the TPT SAM on Ag(111)/mica is a suitable substrate for EBISA, both methods should yield well-defined nanostructures (Figure 1g).

**Figure 2:**
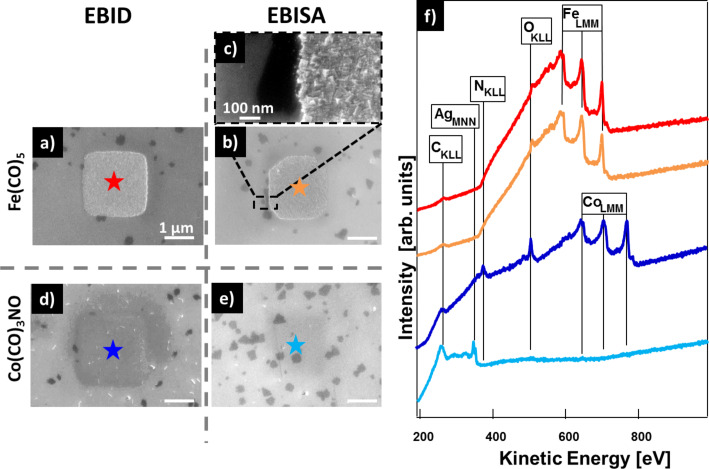
Results of FEBIP experiments followed by autocatalytic growth on a TPT SAM on Ag(111)/mica. All structures were written with *E*_beam_ = 15 kV and *I*_beam_ = 400 pA. (a) SEM image of a 2 × 2 µm^2^ deposit fabricated via EBID + AG with Fe(CO)_5_ (1.04 C/cm^2^ and *t*_AG_ = 3 h 44 min). (b) SEM image of a 2 × 2 µm^2^ deposit fabricated via EBISA + AG with Fe(CO)_5_ (7.80 C/cm^2^ and *t*_AG_ = 4 h 20 min). (c) SEM Blowup image of the structure from (b). (d) SEM image of a 2 × 2 µm^2^ deposit fabricated via EBID + AG with Co(CO)_3_NO (4.68 C/cm^2^ and *t*_AG_ = 3 h 42 min). (e) SEM image of a 2 × 2 µm^2^ deposit fabricated via EBISA + AG with Co(CO)_3_NO (7.80 C/cm^2^ and *t*_AG_ = 4 h 3 min). (f) Local AE spectra recorded at the positions indicated with correspondingly colored stars.

Figure 2a and Figure 2d depict two SE micrographs of 2 × 2 µm^2^ deposits fabricated from, respectively, Fe(CO)_5_ and CO(CO)_3_NO via the EBID + AG process. In Figure 2f the corresponding local AE spectra acquired at the positions indicated with the correspondingly colored stars are plotted. For both precursors selective deposition was observed and no significant unintended co-deposition due to proximity effects [30–31] is visible in the SE micrographs. Local AE spectra of the square fabricated via EBID with Fe(CO)_5_ reveals that the structure consists of basically pure iron (92 atom %), with only minor amounts of carbon and oxygen impurities. The square fabricated from Co(CO)_3_NO consists of cobalt (ca. 45 atom %), oxygen (ca. 45 atom %), and a small amount (<10 atom %) of carbon and nitrogen. Obviously EBID with Co(CO)_3_NO works well on a TPT SAM on Ag(111)/mica. In Figure 2b and Figure 2e two SE micrographs of 2 × 2 µm^2^ deposits, fabricated by EBISA + AG using the same precursors, are depicted. After exposure of Fe(CO)_5_ a clear deposit is visible in Figure 2b. The irradiated 2 × 2 µm^2^ area is not completely covered with iron (purity ca. 90 atom %), as there is no iron at the edges of the square. This process occurs because for EBISA usually a much higher electron dose is necessary to effectively activate the substrate than for EBID. Thus, the edges of the square, which due to the lack of proximity effects are exposed to a lower overall electron dose than the center, are not fully covered. The AG process results in the formation of presumably pure crystalline iron [10,12], as evidenced in the blowup SE image in Figure 2c and the orange spectrum in Figure 2f. In Figure 2e no obvious deposit is visible in the SE micrograph after dosing Co(CO)_3_NO. The faint square shape possibly comes from beam damage. The local AE spectrum acquired in the irradiated area exhibits no Co signals but carbon and silver signals, which can be assigned to the substrate. Consequently, EBISA is feasible on a SAM of TPT molecules with Fe(CO)_5_ but fails on the same substrate with Co(CO)_3_NO. This type of chemical selectivity was reported before on other substrates such as SURMOFs [16–17]. In contrast, on thin layers of porphyrin molecules EBISA was successful with both precursors [14–15]. It is known that electron irradiation of aromatic SAMs causes the cleavage of C–H bonds and, thus, the formation of reactive, that is, activated C species. This is found to be the starting point for the formation of laterally cross-linked CNMs via the formation of C–C linked phenyl species [32]. Considering this, one might suspect that the corresponding activated C species might be also responsible for the dissociation of the Fe(CO)_5_ precursor. In the following we will present a step-by-step investigation to verify this assumption, which will also lead to a better understanding of the temporal behavior of the activated sites. As a first step, one needs to investigate that it is indeed the SAM, and not the underlying Ag substrate, that is active in the EBISA process. Therefore, EBID and EBISA were also conducted on a clean Ag(111)/mica sample without the TPT SAM on top. The results are depicted in Figure S1 (Supporting Information File 1) and can be wrapped up as follows. Selective EBID is possible, and EBISA is not working with both of the precursors on the pristine Ag(111)/mica sample. This evidences that the TPT SAM is crucial for the activation process during EBISA.

**Figure 3:**
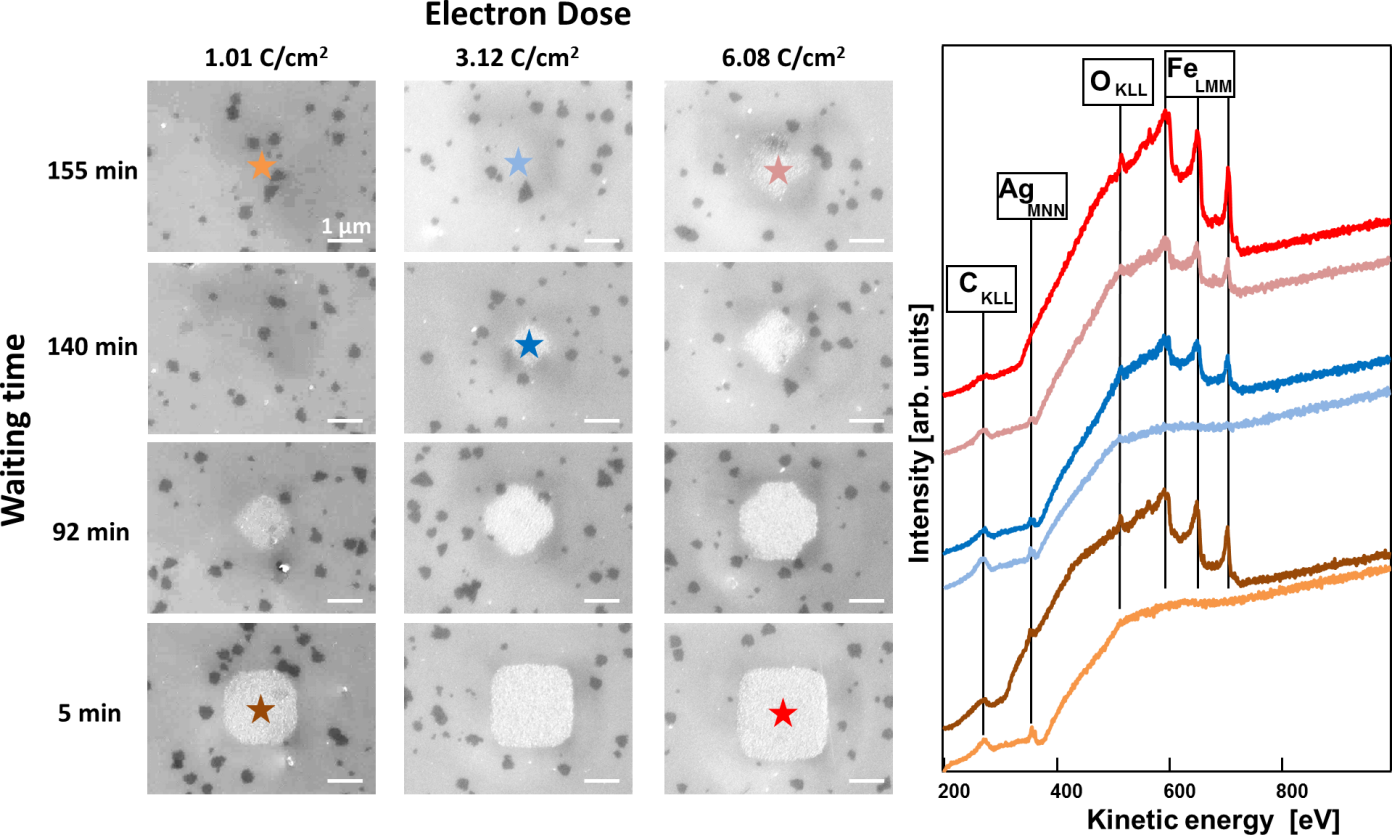
Results of time-dependent EBISA experiments followed by autocatalytic growth on a TPT SAM on Ag(111)/mica. All structures were written with *E*_beam_ = 15 kV, *I*_beam_ = 3 nA, and the same *t*_AG_ = 3 h 29 min. The SEM images of a 2 × 2 µm^2^ deposit fabricated via EBISA + AG with Fe(CO)_5_ can be differentiated regarding electron dose (left column: 1.01 C/cm^2^; medium column: 3.12 C/cm^2^; right column: 6.08 C/cm^2^) and waiting time between electron irradiation and precursor dosage. Local AE spectra recorded at the positions indicated with correspondingly colored stars.

In the next step, the EBISA process will be analyzed on a TPT SAM on Ag(111)/mica that was transformed via electron-induced cross-linking into a CNM beforehand, that is, with a completed cross-linking of the initially electron-activated C bonds. The results of these experiments are depicted in Figure S2 (Supporting Information File 1). While EBID is still working with Fe(CO)_5_ on cross-linked TPT SAM (Figure S2a, Supporting Information File 1), in the EBISA process no iron deposit could be located in the irradiated areas (Figure S2b, Supporting Information File 1). It can be concluded that cross-linked TPT cannot be activated anymore by the electron beam such that Fe(CO)_5_ dissociates at the preirradiated areas. Thus, the predominant C–C bonds in the CNM are either not effectively cleaved during electron exposure or, if they are cleaved, the corresponding sites show no catalytic activity towards the dissociation of Fe(CO)_5_. Considering that, for the cross-linking, C–C bonds are formed from cleaved C–H bonds via electron-induced cross-linking [32], the time interval between these processes seems to play a crucial role for the EBISA process. To be more precise, one anticipates a significant time frame in which the cross-linking between electron beam-activated C atoms occurs based on the latter consideration. To investigate this assumption, we performed experiments in which we varied the waiting time between irradiation of the SAM (Figure 1c) and the following precursor dosage (Figure 1d). In Figure 3 twelve different SE micrographs of 2 × 2 µm^2^ deposits, fabricated from EBISA + AG and Fe(CO)_5_, are depicted. The SE micrographs can be separated in three different electron exposures with doses of 1.01, 3.12, and 6.08 C/cm^2^. For each exposure four SE micrographs are depicted, which only differ in the waiting time between electron irradiation and precursor dosage. All twelve structures were exposed to 3.0 × 10^−7^ mbar Fe(CO)_5_ background pressure for 3 h 29 min. In addition, local AE spectra were acquired at the positions indicated with the correspondingly colored stars. After a comparably short waiting time of 5 min, clear and well-defined square deposits are visible in the SEM image for all three applied doses. Local AE spectra of the structures fabricated from 1.01 and 6.08 C/cm^2^ reveal that the structures consist of iron with very high purity. When the waiting time is increased to 92 min, for all electron doses, the irradiated square areas are apparently not fully covered by deposits. For the highest electron dose of 6.08 C/cm^2^ just small areas at the edges of the squares are not covered. The area of the uncovered parts increases with decreasing electron dose. At 1.01 C/cm^2^ the structure rather has the shape of a rhomb than that of a square. After further increasing the waiting time to 140 min, no iron deposit is visible for a dose of 1.01 C/cm^2^. At 3.12 C/cm^2^ a small amount of iron is deposited, whereas the covered area is further decreasing compared to 92 min waiting time. The same is true for the structure fabricated with 6.08 C/cm^2^. When the waiting time is increased to 155 min for 1.01 and 3.12 C/cm^2^, no iron is visible within the irradiated area and no iron is detected in the corresponding local AE spectra. Only at the highest dose of 6.08 C/cm^2^ a small amount of iron is detected in the local AE spectra. The apparently deposit-covered area is further decreasing compared to shorter waiting times. The entire set of the experiment with different values for the waiting time is documented in Supporting Information File 1, Figure S3 (for 1.01 C/cm^2^), Figure S4 (for 3.12 C/cm^2^), and Figure S5 (for 6.08 C/cm^2^). The latter data reveals that the effect apparently directly correlates with the waiting time.

How can this apparent time dependence of the EBISA process, which has been observed for the first time, be explained? Apparently, the amount of activated sites that lead to a dissociation of Fe(CO)_5_ is decreasing over time. The longer the waiting time between electron irradiation and precursor dosage, the higher is the degree of deactivation of the activated sites. If one assumes that the number of active sites increases with electron dose, the dose-dependent behavior observed in Figure 3 can be derived. It was reported that electron irradiation of aromatic SAMs leads to the cleavage of C–H bonds and the formation of cross-linked CNMs via the formation of C–C bonds [32]. We thus propose that the cleavage of C–H bonds creates reactive sites that are responsible for the dissociation of Fe(CO)_5_. These active sites might be deactivated over time by reaction with neighboring molecules to form C–C bonds, that is, the cross-linking process. If it takes a comparatively long time until all reactive species find a suitable reaction partner, the EBISA deactivation process is anticipated to occur on the same time scale. Another possibility might be that some of the reactive species just do not find a suitable reaction partner due to sterical reasons and are deactivated over time either by residual gases in UHV or by an electron quenching effect via the surface or neighboring molecules [32].

EBISA should also work with electrons of rather low energy fabricated by a flood gun instead of the focused electron beam. This macroscopic process is schematically depicted in Figure S6 (Supporting Information File 1). If the precursor gas dosage takes place directly after electron irradiation (100 eV, 120 mC/cm^2^) the whole surface of the cross-linked SAM is afterwards covered with clean iron nanocrystals (Figure S6d,e in Supporting Information File 1). The insulating ultrathin CNM is covered with a thin layer of conductive ferromagnetic iron crystals. By this process laterally functionalized 2D CNMs can be fabricated.

**Figure 4:**
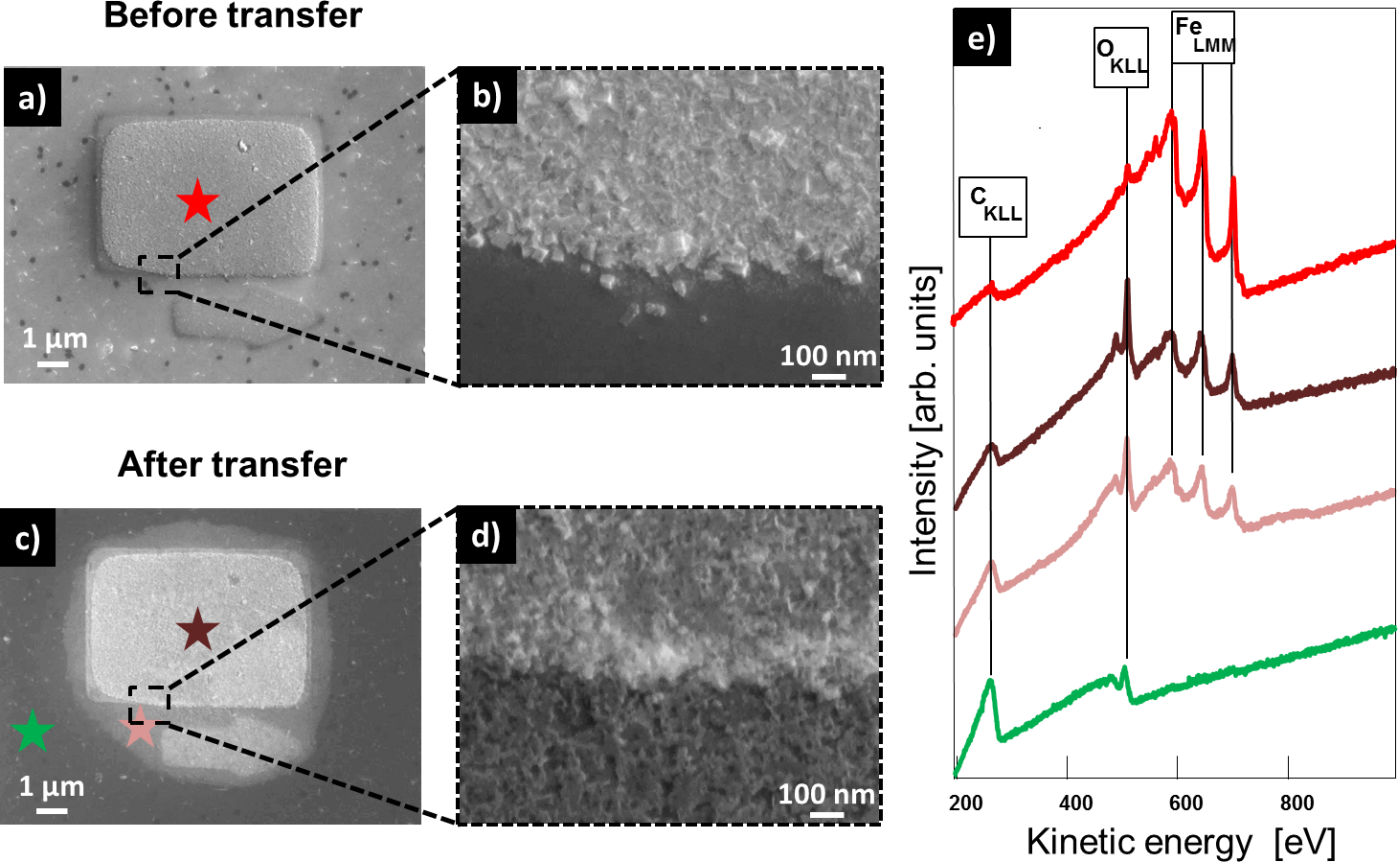
Transfer of a CNM with a Fe structure on top onto a SiO_2_ sample. (a) SEM image of a Fe structure fabricated with EBID on a TPT SAM on Ag(111)/mica (beam parameters 15 kV, 400 pA, electron dose: 0.93 C/cm^2^, and AG time: 4 h 5 min). (b) Blowup image of the structure depicted in (a). (c) SEM image of the Fe structure depicted in (a) after the transfer to bulk SiO_2_. (d) Blowup image of the structure depicted in (c). (e) Local AES spectra recorded at the positions indicated with the correspondingly colored stars.

In a recent study, it was reported that it is possible to fabricate nanostructures on a TPT/Au system and transfer the nanostructures on top of the CNM onto arbitrary substrates. In these experiments the nanostructures retained their shape and did not undergo any changes, aside from oxidation, during the transfer process [21]. In this work, we studied a new system by using TPT SAMs on Ag instead of the analogue SAMs on Au. The main difference caused by changing the substrate is the different wet-chemical etching solution that is necessary for the lift-off process. For etching of the Au layer a solution of KI/I_2_/H_2_O is used, whereas for the dissolution of Ag a solution of Fe(NO_3_)_3_ is necessary. The SAM was cross-linked into a CNM by using a flood gun employing 100 eV electrons and an electron dose of 60 mC/cm^2^ after the EBID structures were fabricated. Before removing the Ag layer by putting it into a 1 M Fe(NO_3_)_3_ solution for 24 h, the sample was protected by a 400 nm thick layer of poly(methyl methacrylate) (PMMA). In a next step, the CNM/EBID/PMMA hybrid structure was transferred onto a SiO_2_ substrate. Finally, the PMMA was dissolved in acetone. The results for this transfer process with EBID structures fabricated from Fe(CO)_5_ are depicted in Figure 4. The SE micrograph in Figure 4a depicts a several micrometers large marker structure fabricated via EBID + AG using Fe(CO)_5_. Through the AG process iron nanocrystals are formed (Figure 4b). After the transfer onto a bulk SiO_2_ substrate the same structure could be relocated. However, the appearance in the SEM image of the structure changed (Figure 4c). A bright circular shape is located around the structure. Furthermore, no clear iron nanocrystals are visible anymore in the corresponding blowup image depicted in Figure 4d. Before the transfer, the structure consisted of iron nanocrystals with very high purity (87 atom %) as shown in the AE spectrum in Figure 4e. After the transfer, the structure was oxidized (54 atom % Fe) due to exposure to the ambient. The bright circular feature around the structure also consists of iron oxide, whereas no iron is detected on the rest of the surface. Only carbon and oxygen resulting from the CNM and the underlying SiO_2_ substrate can be found. Compared to the transfer of a SAM/CNM grown on a layer of Au, where the iron structures remain completely intact aside from oxidation [21], the transfer of a SAM/CNM grown on Ag induces more significant changes to the deposit. One main difference of the transfer process is that for the dissolution of the Ag layer the sample is exposed to a Fe(NO_3_)_3_ solution overnight, whereas the Au layer can be etched away with a KI/I_2_/H_2_O solution within 10 min. Apparently the Fe(NO_3_)_3_ solution is responsible for the observed effect. It might be possible that the Fe(NO_3_)_3_ solution is able to diffuse through possible ruptures in the CNM or the PMMA layer. This diffusion process might lead to the dissolution/reduction of the iron structures [33]. The bright circular shape around the structure depicted in Figure 4c can be explained by an incomplete dissolution process. After removing the sample from the Fe(NO_3_)_3_ solution this type of deformed structure remains.

**Figure 5:**
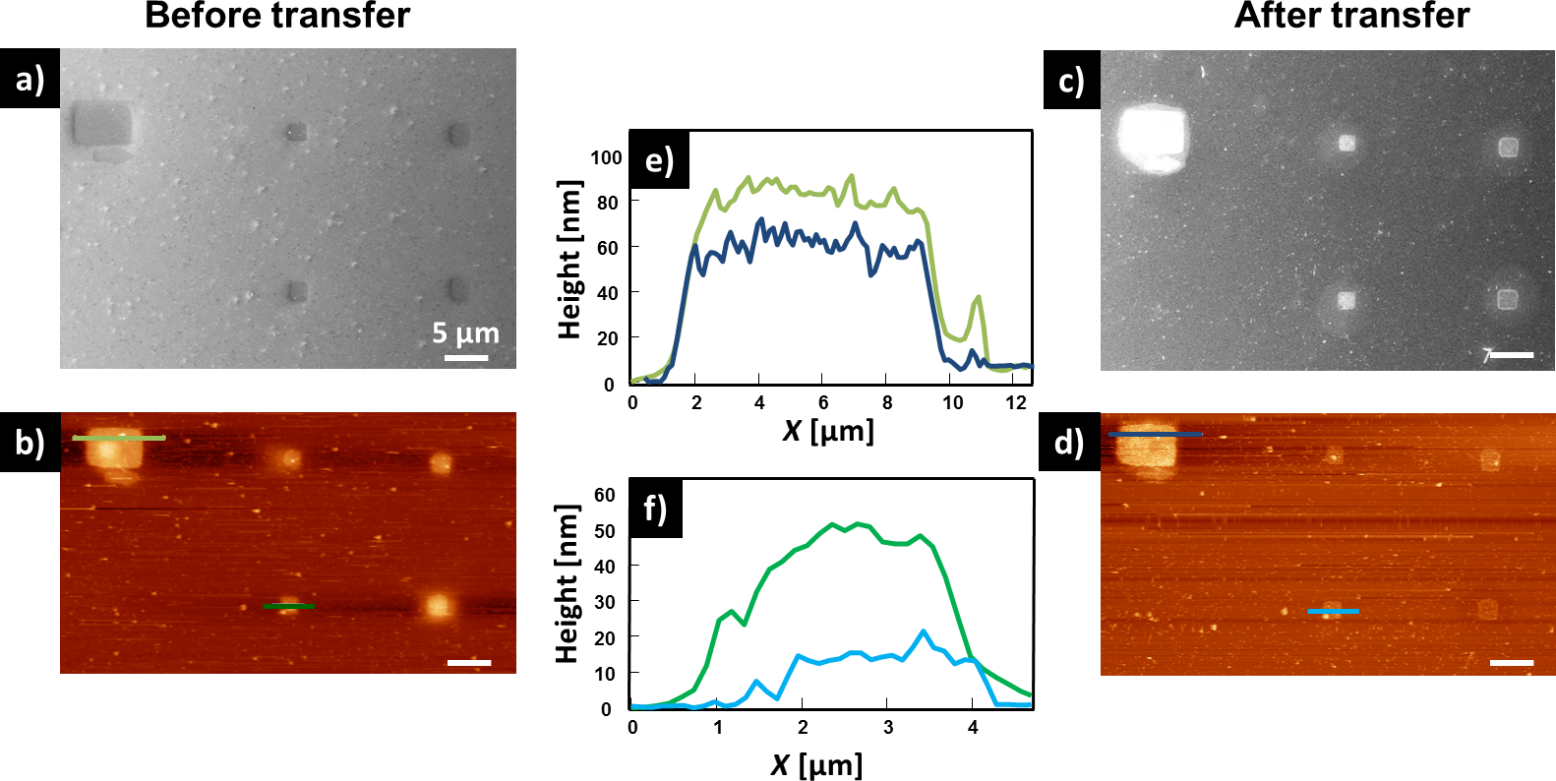
Transfer of a CNM with Fe structures on top onto a SiO_2_ sample. (a) SEM image of the Fe structures fabricated with EBID on a TPT SAM on Ag(111)/mica. (b) AFM image of the structures depicted in (a). (c) SEM image of the Fe structures depicted in (a) after the transfer to bulk SiO_2_. (d) AFM image of the Fe structures after the transfer to bulk SiO_2_. (e) Height profile of the larger marker structure before and after the transfer. (f) Height profile of a smaller square structure before and after the transfer.

To gain further insight into the effect of the Fe(NO_3_)_3_ solution onto the transfer process, AFM measurements were done before and after the transfer. In Figure 5a and Figure 5b, SEM and AFM images, respectively, of five different iron structures are depicted. One larger structure, which acts as a marker structure (the same one as in Figure 4a), and four 2 × 2 µm^2^ squares can be seen. The structures are distributed over an area of 50 × 40 µm^2^.The marker structure (7 × 5 µm^2^) is the largest transferred iron structure. In Figure 5c and Figure 5d SEM and AFM images, respectively, of the same five structures after the transfer onto a bulk SiO_2_ substrate are depicted. The large marker structure decreased in height from 80 nm before the transfer to 60 nm after the transfer (Figure 5e). The bright circular feature around the structure exhibits a height of roughly 15 nm (Figure S7, Supporting Information File 1). For the smaller structures the height decrease was even more pronounced. One example is depicted in Figure 5f. This structure had initially a height of roughly 50 nm, whereas after the transfer the height decreased to ca. 15 nm. This height reduction and the bright circular shape around the structures after the transfer process indicate that the iron structures are slowly dissolved during exposure to the Fe(NO_3_)_3_ solution.

**Figure 6:**
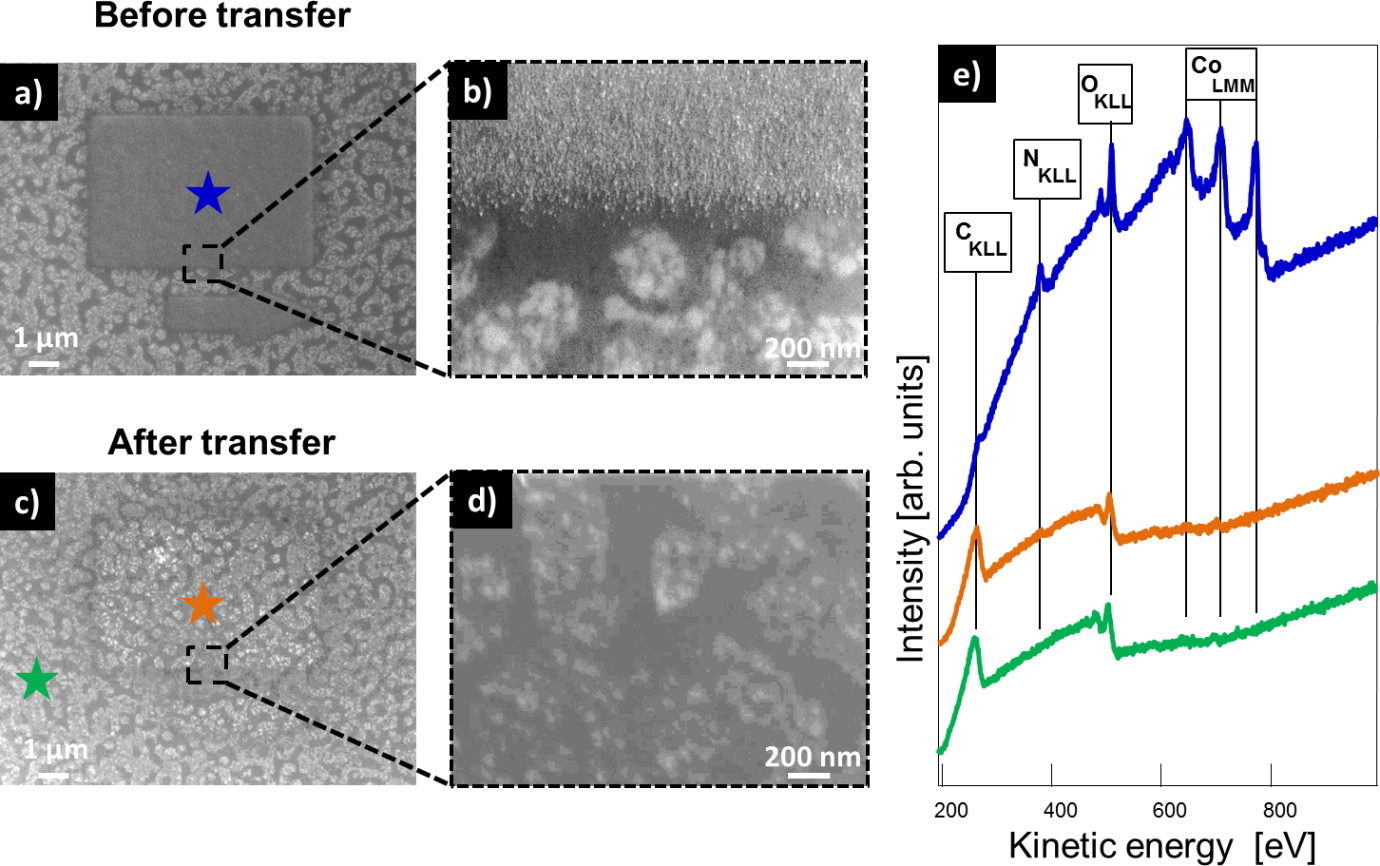
Transfer of a CNM with a cobalt oxide structure on top onto a SiO_2_ sample. (a) SEM image of a cobalt oxide structure fabricated with EBID on a TPT SAM on Ag(111)/mica (beam parameters 15 kV, 3 nA, electron dose: 0.93 C/cm^2^, and AG time: 3 h 52 min). (b) Blowup image of the structure depicted in (a). (c) SEM image of the cobalt oxide structure depicted in (a) after the transfer to bulk SiO_2_. (d) Blowup image of the structure depicted in (c). (e) Local AES spectra recorded at the positions indicated with correspondingly colored stars.

The same transfer process was also performed with EBID structures fabricated from Co(CO)_3_NO. The results are depicted in Figure 6. Figure 6a shows a SEM image of a marker structure fabricated via EBID + AG using Co(CO)_3_NO. A comparable structure fabricated on the same substrate exhibits a height of 60 nm (Figure S8, Supporting Information File 1). The resulting deposit consists of small cobalt oxide particles, interpreted on the basis of the corresponding blowup image (Figure 6a) and the local AE spectrum (Figure 6e). Only minor impurities of carbon and nitrogen are detected. In the SEM image recorded after the transfer onto a bulk SiO_2_ substrate only a faint shape of the original structure is visible (Figure 6c). Furthermore, no cobalt oxide particles are present anymore in the blowup image depicted in Figure 6d. In addition, no cobalt signal was detected in the AE spectrum recorded in the area where the deposit was located initially. The AE signal is similar to the signal of the pure CNM on SiO_2_. It only exhibits carbon and oxygen signals. The cobalt oxide structure has completely vanished after the transfer process. Apparently, the Fe(NO_3_)_3_ solution dissolves the cobalt oxide structures along with the silver substrate.

**Figure 7:**
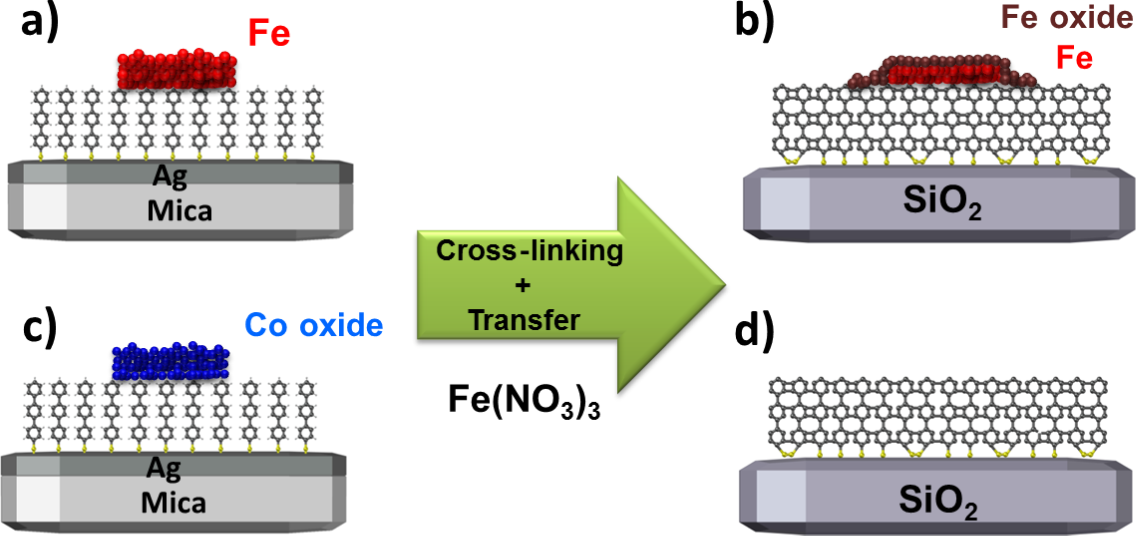
Schematic result of the transfer process. (a) Fe structure on top of a TPT SAM on a Ag(111)/mica sample. (b) Fe structure after cross-linking the SAM and transferring the sample onto bulk SiO_2_. The height decreases, and the structure is oxidized. (c) Cobalt oxide structure on top of TPT on a Ag(111)/mica sample. (d) After cross-linking the SAM and transferring the sample onto bulk SiO_2_, the cobalt oxide structure has vanished completely.

In summary, the results from the transfer process of EBID structures on a SAM grown on silver indicate that, in the case of iron, the structures are decreasing in height. Also a bright circular shape, most probably caused by diffusion and dissolution or reduction [33] in the Fe(NO_3_)_3_ solution, around the structure is formed. For EBID structures fabricated from Co(CO)_3_NO it is obvious that the Fe(NO_3_)_3_ solution dissolves the cobalt oxide particles completely. This outcome of the transfer process is schematically depicted in Figure 7. With this we want to emphasize the importance of the underlying substrate and the corresponding chemistry to dissolve the latter in such transfer processes. Maybe other EBID deposits fabricated from Au [34] or Ag [35] precursors are more suitable as they might be more inert towards the Fe(NO_3_)_3_ solution. Also, a different type of lift-off mechanism might be more successful regarding the transfer of iron and cobalt oxide structures.

## Conclusion

In conclusion, we investigated two different FEBIP methods, namely EBID and EBISA, on a SAM (TPT) grown on silver. Fe(CO)_5_ and Co(CO)_3_NO were studied as precursors. We could show that EBID is successful with both precursors, whereas EBISA only works with Fe(CO)_5_. This type of chemical selectivity was already reported in previous studies on SURMOFs [16–17]. For SAMs, we assume that the active species that leads to the dissociation of Fe(CO)_5_ is formed upon electron-induced cleavage of C–H bonds, that is, an activated C species. Interestingly, we observed a strong time dependence of the EBISA process. The active species seem to get deactivated with time so that no iron will be deposited anymore. This type of deactivation process was not reported before [10,12,14–17]. The mechanism for the time-dependent deactivation process remains somewhat speculative. We narrow it down to two possibilities. First, it might be that the cross-linking is a competing effect, that is, activated carbon atoms are deactivated by cross-linking and, thus, no longer available to decompose subsequently dosed precursor molecules. This would also mean that the cross-linking process is rather slow, that is, it completes in a time frame of an hour or more, depending on the applied electron dose. The second route is proposed to be due to the deactivation via interaction of active sites with residual gas molecules. At this point, it should be noted that a mixture of both effects appears to be most likely. However, the quantitative contribution is not known. As it was reported that free-standing hybrid structures consisting of metallic nanostructures on top of CNMs can be fabricated by transferring them from a SAM/CNM grown on Au, we expanded and investigated this process by using a SAM/CNM grown on Ag. In the case of iron structures, diffusion processes caused by the Fe(NO_3_)_3_ solution, which is used to dissolve the silver layer, lead to a significant height decrease and a change in the appearance of the FEBIP deposits. Structures consisting of cobalt oxide are completely dissolved during the transfer process. Hence, we emphasize that for a successful fabrication of such hybrid structures a suitable combination of substrate for the growth of the SAM, etching solution, and metallic nanostructures needs to be considered. Especially regarding the large amount of possible precursors in FEBIP [4], for each type of metallic nanostructure a suitable transfer mechanism needs to be investigated and the interactions during the transfer process need to be analyzed.

## Experimental

The employed Ag substrates (Georg Albert PVD) consist of a 300 nm thick layer of Ag(111) on mica. Prior to SAM preparation, the substrates were soaked for 20 min and rinsed thoroughly in *N*,*N*-dimethylformamide (DMF, Sigma-Aldrich, 99.8%) and then in ethanol (VWR BHD CHEMICALS, 99.9%). The precursor 1,1′,4′,1′′-terphenyl-4-thiol (TPT) (Sigma-Aldrich, 97%) was sublimated before use. Preparation of SAMs/Ag follows the wet method for the analogous SAMs/Au as described elsewhere [26]. Silver substrates were immersed in a ca. 1 mM TPT solution under argon environment for 24 h at 70 °C. Then, a repetition of rinsing in DMF and ethanol was applied to the samples in order to remove physically absorbed TPT molecules. The samples were consequently dried by a stream of nitrogen gas and preserved in argon environment until use in FEBID experiments. The FEBIP experiments were performed in a commercial UHV system (Multiscanlab, Omicron Nanotechnology, Germany) with a base pressure of *p* < 2 × 10^−10^ mbar. The main component of the system is a UHV-compatible electron column (Leo Gemini) for scanning electron microscopy (SEM, nominal resolution better than 3 nm) and a local AES using a hemispherical energy analyzer. Fe(CO)_5_ was purchased from ACROS Organics. Co(CO)_3_NO was purchased from abcr GmbH & Co. KG. The quality of the precursor gas was analyzed with a quadrupole mass spectrometer in a dedicated gas analysis chamber (base pressure below 2 × 10^−9^ mbar). The precursor gas was dosed through a nozzle with an inner diameter of 3 mm and a distance of approximately 12 mm to the sample surface. Based on simulations using GIS Simulator (version 1.5) [36], the local pressure increase on the sample surface was calculated to approximately 30×. For a fixed background pressure of 3.0 × 10^−7^ mbar, this corresponds to a local pressure at the surface of about 9 × 10^−6^ mbar [37]. All electron exposures for SEM and lithography were performed at a beam energy of 15 kV and probe currents of 400 pA and 3 nA, respectively. The lithographic processes were controlled via a self-developed lithography application based on LabView 8.6 (National Instruments) and a high-speed DAC PCIe card (M2i.6021-exp, Spectrum GmbH, Germany). SEM images were acquired with SmartSEM (Zeiss) and are shown with minor contrast and brightness adjustments only. For Auger electron spectroscopy the electron beam of the SEM was used as ionization source at an energy of 15 keV and a beam current of 3.0 nA. Spectra were recorded with a hemispherical electron energy analyzer (EA125, Omicron Nanotechnology) and EIS 2.4.24.97 (Omicron Nanotechnology). Data processing was performed with Igor Pro 6.22A (Wavemetrics). The AFM experiments were performed with a JPK NanoWizard 4 by using non-contact mode. Cross-linking of SAMs into CNMs was achieved by using electron flood guns employing 100 eV electrons and an electron dose of 60 mC/cm^2^. Before starting the transfer process, the cross-linked CNMs were spin coated with a protecting layer of PMMA with an overall thickness of ca. 400 nm. First, a layer of low-molecular-weight PMMA (35 ku), then, a layer of high-molecular-weight PMMA (996 ku) were spin cast each for 1 min at 4000 rpm and cured on a hot plate at 363 K for 5 min. The 300 nm thick silver layer was removed after immersing the sample in a 1 M Fe(NO_3_)_3_ solution (ACROS Organics). The PMMA/CNM is then transferred into pure water in order to remove iron contamination. In the next step the PMMA/CNM is transferred onto a bulk SiO_2_ substrate. After drying the sample overnight, the PMMA layer is dissolved by soaking the sample in acetone for 1 h.

## Supporting Information

Supporting Information File 1 contains further information on EBID and EBISA on Ag(111)/mica surfaces and CNMs, the time dependence of the EBISA process, the “whole-area” EBISA approach, and additional AFM images.

File 1 Additional experimental data.
