# Supplementary material for: Exploring the fabrication and transfer mechanism of metallic nanostructures on carbon nanomembranes via focused electron beam induced processing
Source: Beilstein J Nanotechnol. 2021 Apr 7;12:319–29. doi: 10.3762/bjnano.12.26 (PMC8042486; doi:10.3762/bjnano.12.26)
Supplement: File 1 — Additional experimental data. [file Beilstein_J_Nanotechnol-12-319-s001.pdf]

BEILSTEIN JOURNAL OF NANOTECHNOLOGY

Supporting Information

for

Exploring the fabrication and transfer mechanism of metallic nanostructures on carbon nanomembranes via focused electron beam induced processing

Christian Preischl, Linh Hoang Le, Elif Bilgilisoy, Armin Gölzhäuser
and Hubertus Marbach

Beilstein J. Nanotechnol. **2021,** *12,* 319–329. doi:10.3762/bjnano.12.26

Additional experimental data

License and Terms: This is a supporting information file under the terms of the Creative Commons Attribution License (https://creativecommons.org/licenses/by/4.0). Please note that the reuse, redistribution and reproduction in particular requires that the author(s) and source are credited and that individual graphics may be subject to special legal provisions.
The license is subject to the *Beilstein Journal of Nanotechnology* terms and conditions: (https://www.beilstein-journals.org/bjnano/terms)

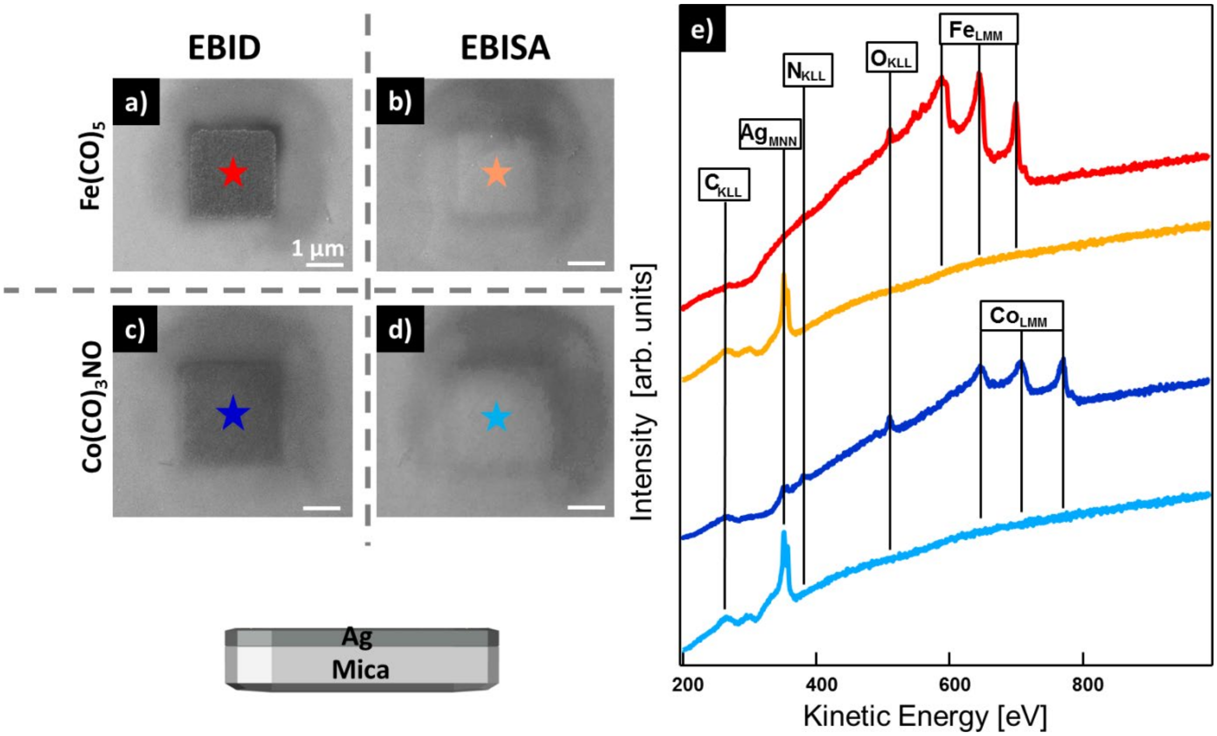

Figure S1: Results of FEBIP experiments followed by autocatalytic growth on Ag(111)/mica. All structures were written with E_{beam} = 15 kV and I_{beam} = 3 nA. (a) SEM image of a 2 × 2 µm^2 deposit fabricated via EBID + AG with $Fe(CO)_5$ (4.7 C/cm^2 and t_{AG} = 2 h 26 min). (b) SEM image of a 2 × 2 µm^2 deposit fabricated via EBISA + AG with $Fe(CO)_5$ (10.1 C/cm^2 and t_{AG} = 2 h 41 min). (c) SEM image of a 2 × 2 µm^2 deposit fabricated via EBID + AG with $Co(CO)_3NO$ (4.7 C/cm^2 and t_{AG} = 2 h 24 min). (d) SEM image of a 2 × 2 µm^2 deposit fabricated via EBISA + AG with $Co(CO)_3NO$ (10.1 C/cm^2 and t_{AG} = 2 h 47 min). (e) Local AE spectra recorded at the positions indicated with the correspondingly colored stars.

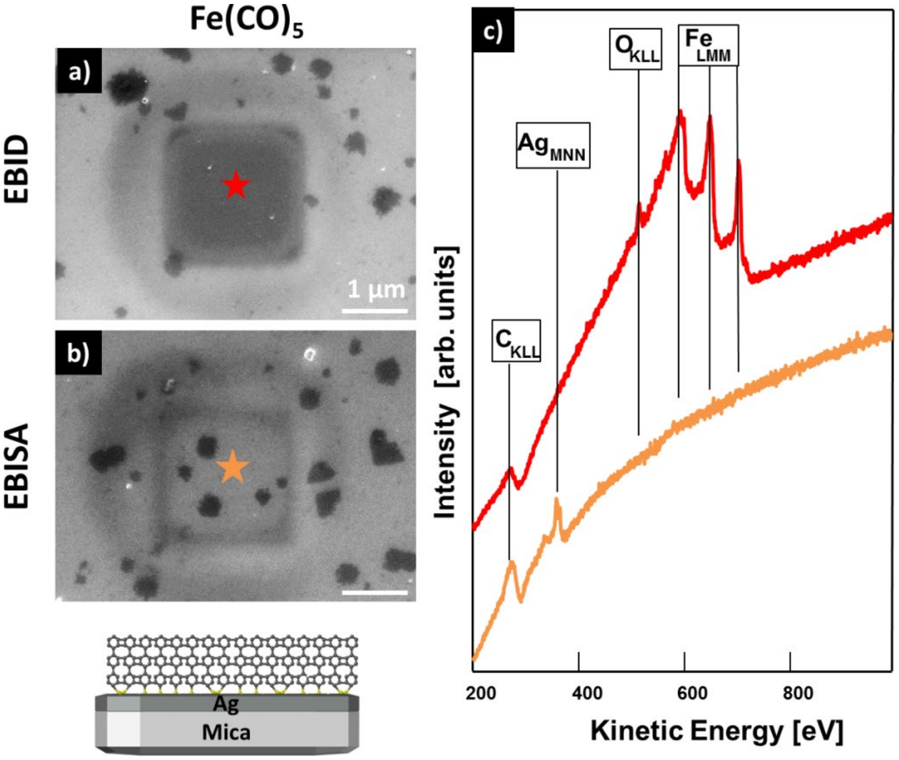

Figure S2: Results of FEBIP experiments followed by autocatalytic growth on a CNM on Ag(111)/mica. All structures were written with E_{beam} = 15 kV and I_{beam} = 3 nA. (a) SEM image of a 2 × 2 µm^2 deposit fabricated via EBID + AG with $Fe(CO)_5$ (7.8 C/cm^2 and t_{AG} = 3 h 38 min). (b) SEM image of a 2 × 2 µm^2 deposit fabricated via EBISA + AG with $Fe(CO)_5$ (7.8 C/cm^2 and t_{AG} = 4 h 2 min). (c) Local AE spectra recorded at the positions indicated with the correspondingly colored stars.

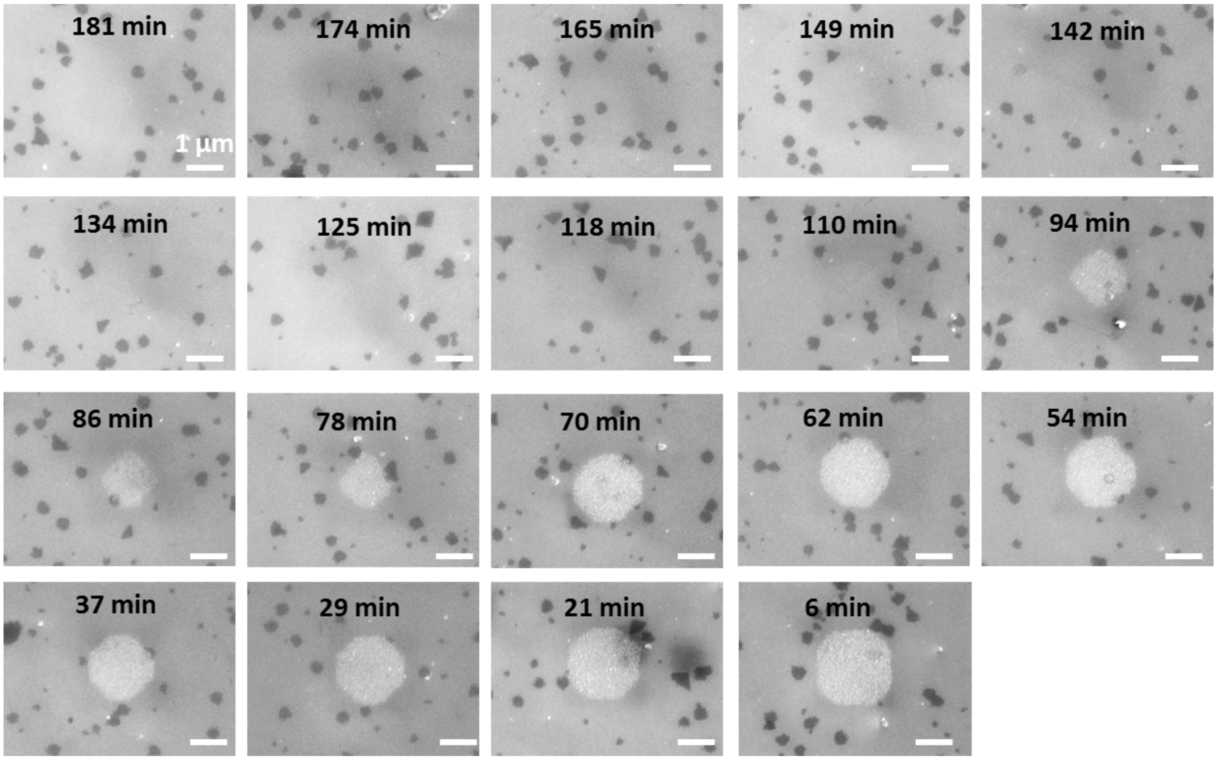

Figure S3: Results of time-dependent EBISA experiments followed by autocatalytic growth on a TPT SAM on Ag(111)/mica. All structures were written with E_{beam} = 15 kV, I_{beam} = 3 nA, and the same t_{AG} = 3 h 29 min. SEM images of 2 × 2 µm^2 deposits fabricated via EBISA + AG with $Fe(CO)_5$ (electron dose: 1.01 C/cm^2). The waiting time between electron irradiation and precursor dosage is indicated for each structure.

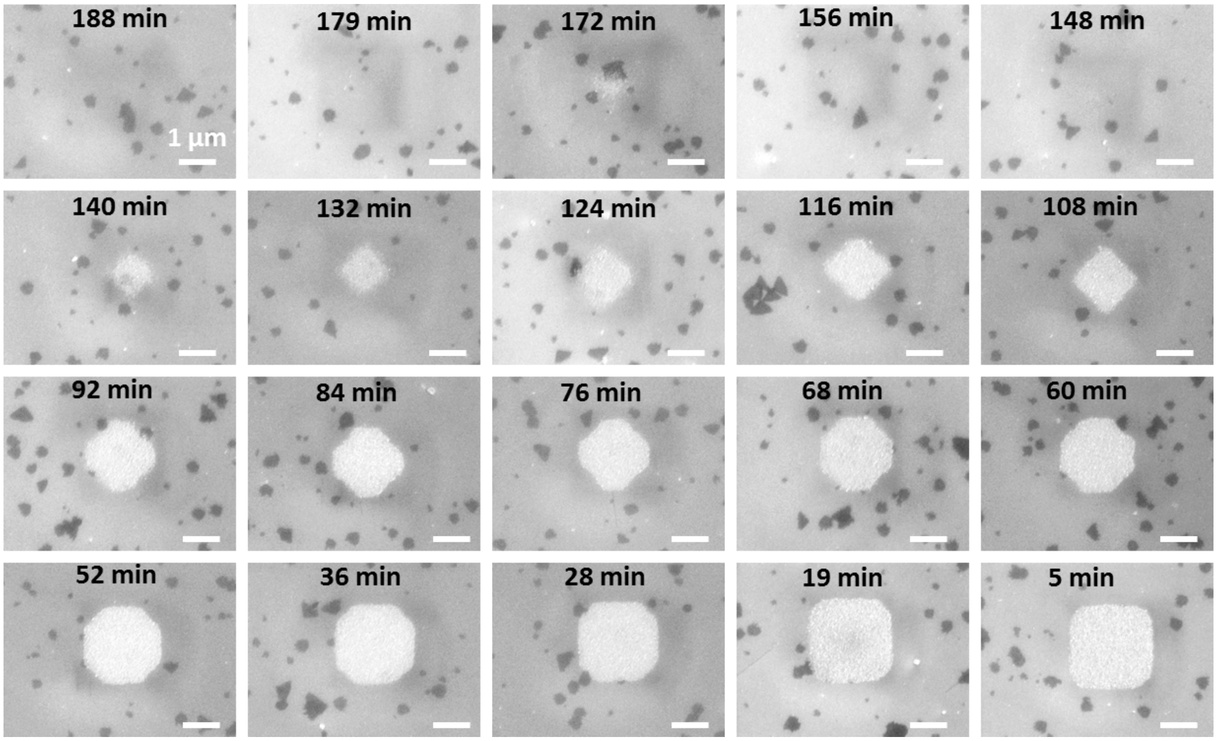

Figure S4: Results of time-dependent EBISA experiments followed by autocatalytic growth on a TPT SAM on Ag(111)/mica. All structures were written with E_{beam} = 15 kV, I_{beam} = 3 nA, and the same t_{AG} = 3 h 29 min; SEM images of 2 × 2 µm^2 deposits fabricated via EBISA + AG with $Fe(CO)_5$ (electron dose: 3.12 C/cm^2). The waiting time between electron irradiation and precursor dosage is indicated for each structure.

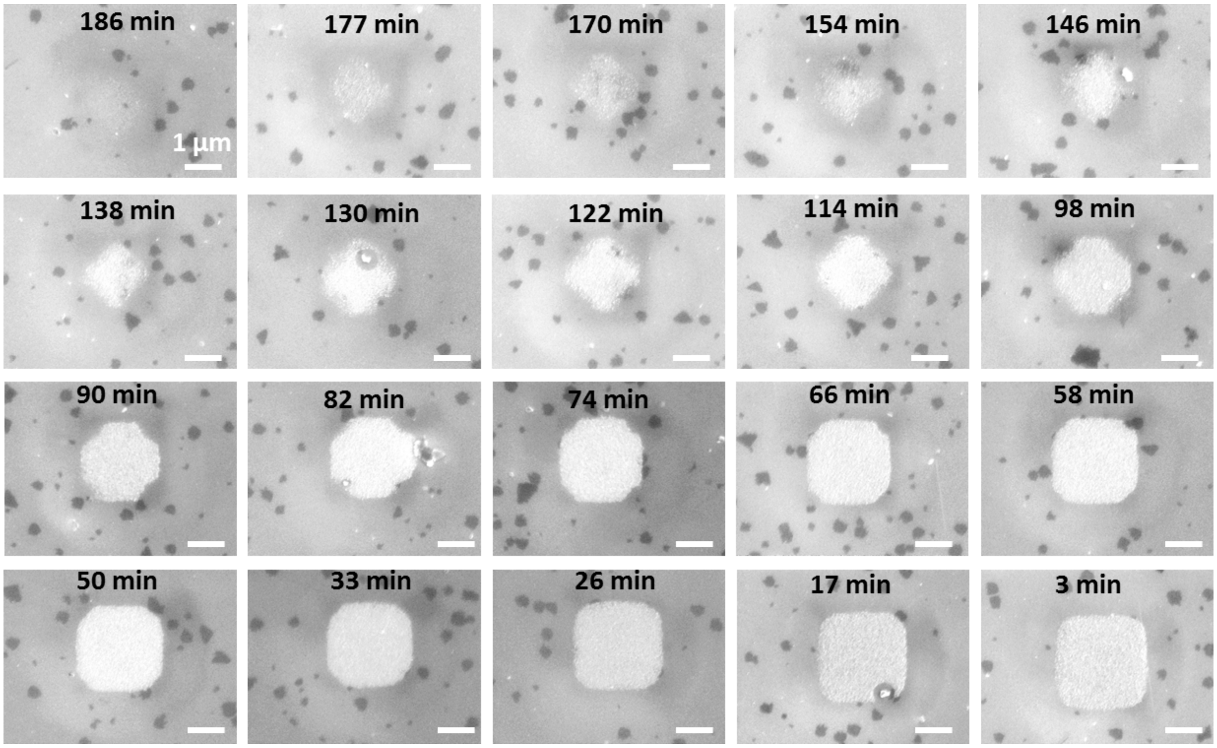

Figure S5: Results of time-dependent EBISA experiments followed by autocatalytic growth on a TPT SAM on Ag(111)/mica. All structures were written with E_{beam} = 15 kV, I_{beam} = 3 nA, and the same t_{AG} = 3 h 29 min. SEM images of 2 × 2 µm^2 deposits fabricated via EBISA + AG with $Fe(CO)_5$ (electron dose: 6.08 C/cm^2). The waiting time between electron irradiation and precursor dosage is indicated for each structure.

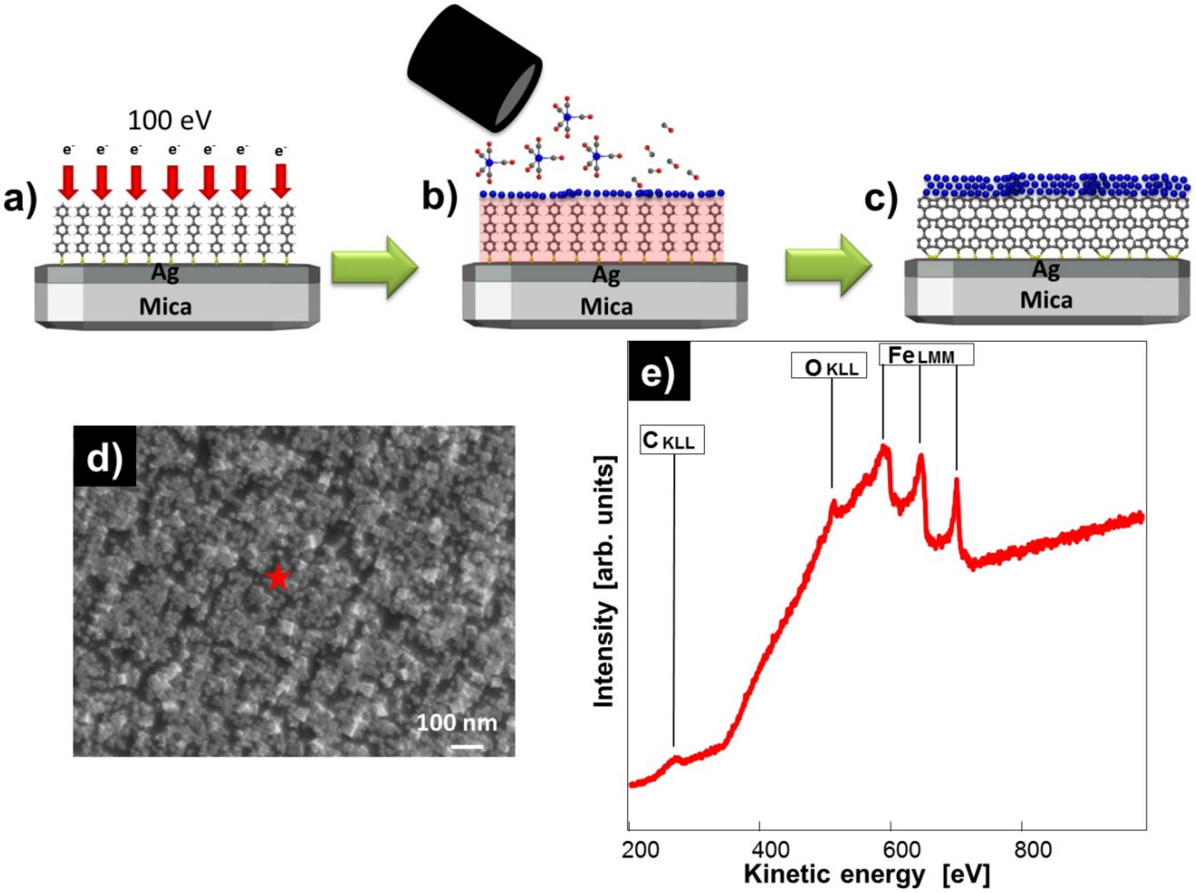

Figure S6: EBISA-like process with low-energy electrons. (a) Irradiation of a TPT SAM on Ag(111)/mica (100 eV; 120 mC/cm^2) by a flood gun. (b) Dosage of the $Fe(CO)_5$ precursor for 4 h. The active species in the SAM are indicated by the red rectangle. (c) Cross-linked CNM with Fe nanocrystals on top. (d) SEM image of the cross-linked CNM with Fe nanocrystals on top. (e) Local AE spectrum recorded at the position indicated by the colored star.

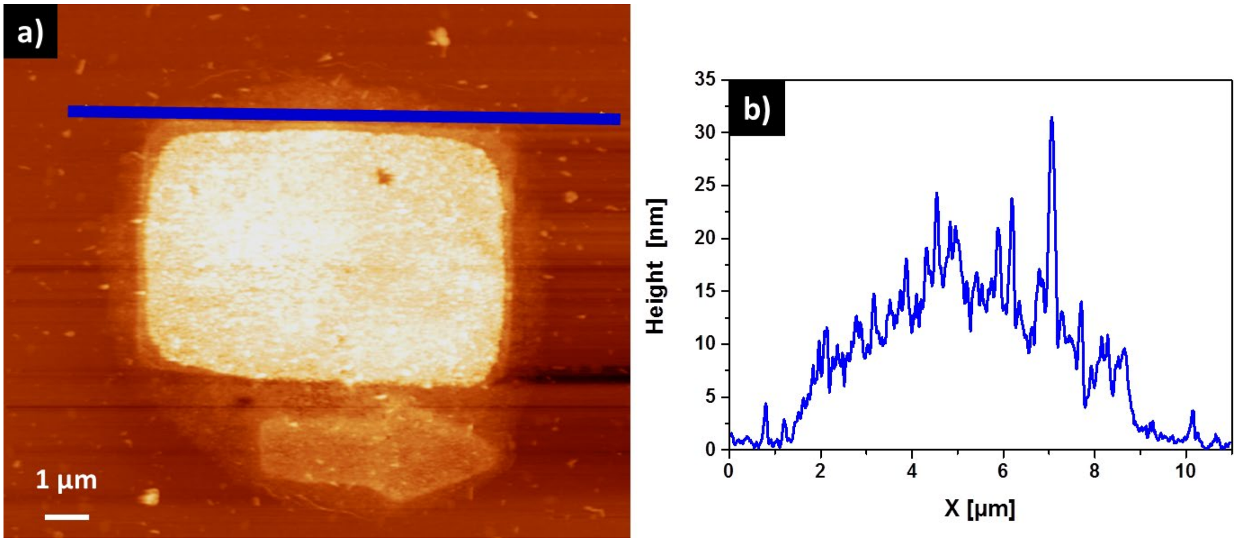

Figure S7: (a) AFM image of the Fe structure shown in Figure 4c of the main manuscript. (b) Height profile of the bright circular feature around the Fe structure

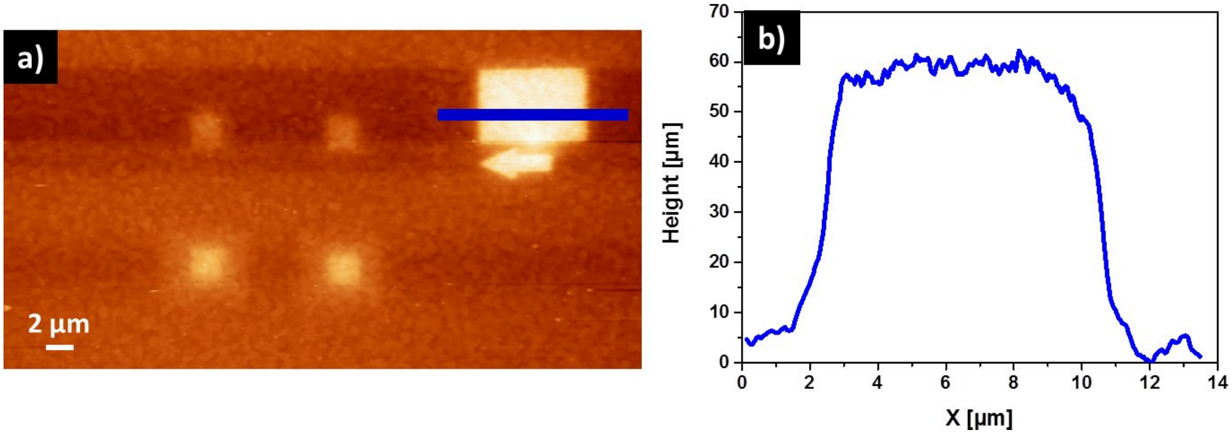

Figure S8: (a) AFM image of five different cobalt oxide structures before the transfer process. (b) Height profile of the large marker structure
